# Establishment and characterization of three stable Basal/HER2-positive breast cancer cell lines derived from Chinese breast carcinoma with identical missense mutations in the DNA-binding domain of TP53

**DOI:** 10.1186/s12935-018-0617-9

**Published:** 2018-08-17

**Authors:** Fei Zhou, Yanhua Zhang, Xiufang Xu, Jingfeng Luo, Fang Yang, Linbo Wang, Shuduo Xie, Jihong Sun, Xiaoming Yang

**Affiliations:** 1 Department of Radiology, Sir Run Run Shaw Hospital, Zhejiang University School of Medicine, Hangzhou, Zhejiang China; 2 Department of Pathology, Sir Run Run Shaw Hospital, Zhejiang University School of Medicine, Hangzhou, Zhejiang China; 3 Department of Medical Imagine, Hangzhou Medical College, Hangzhou, Zhejiang China; 4 Department of Surgical Oncology, Sir Run Run Shaw Hospital, Zhejiang University School of Medicine, Hangzhou, Zhejiang China; 5 Image-Guided Bio-Molecular Intervention Research, Department of Radiology, University of Washington School of Medicine, Seattle, WA USA

**Keywords:** Basal/HER2-positive, Breast cancer cell lines, Invasive ductal breast carcinoma, Epithelial cell, STR

## Abstract

**Background:**

Basal/human epidermal growth factor receptor (HER)2-positive (HER2+) breast cancer is resistant to monoclonal antibody (herceptin) treatment. There are currently only three basal/HER2+ breast cancer cell lines available, but they are not from Chinese populations.

**Methods:**

Three immortalized cell lines (ZJU-0327, ZJU-0725, and ZJU-1127) were established from invasive ductal breast carcinoma tissue of two patients treated by surgical resection at our center. The cell lines were characterized in terms of histology, therapeutic response, and biomarker expression. Their tumorigenic potential was evaluated in an athymic nude (BALB/C nu) mouse xenograft model. Cell authentication testing by the techniques of short tandem repeat.

**Results:**

ZJU-0327, ZJU-0725, and ZJU-1127 cell lines were maintained for more than 110 passages in vitro. The cells grew as monolayers; showed typical epithelial morphology and ultrastructure; were polyploid; had doubling times of 18, 57.5, and 18 h, respectively; had a near-tetraploid (ZJU-0327 and ZJU-1127) or aneuploid (ZJU-0725) karyotype with structural aberrations and tumor protein 53 mutation; insensitive to chemotherapeutic drugs and/or radiation; show high invasiveness and tumorigenicity in mice; and had no mycoplasma contamination. The cell lines were basal/HER2+, expressed cluster of differentiation, and were associated with poor prognosis. Cell authentication testing by the American Type Culture Collection confirmed the human origin of the cell lines, which did not match those in existing databases.

**Conclusions:**

The three novel basal/HER2+ breast cancer cell lines recapitulating the malignant characteristics of the parent tumor’s, and can be useful for clarifying the molecular pathogenesis of basal/HER2+ breast cancer.

## Background

Breast cancer is the first occurring incidence overall and is the second leading cause of death in the United States in 2017 [1]. It is also major threat to women’s health in China. Over the past decade, several pathological and immunohistochemical sub-classifications have been proposed to better characterize the numerous and heterogeneous molecular features of hormone receptor-positive and triple-negative breast cancer at the clinical level; however, these have not included HER2+ breast cancer. High rates of inherent resistance to treatment with the monoclonal antibody trastuzumab (herceptin) are common among HER2 gene-amplified breast carcinomas in both metastatic and adjuvant settings [2]; moreover, basal/HER2+ patients have the worst disease-free and overall survival among HER2+ subtypes [3], although the molecular basis for these observations is not well understood.

Establishing cell lines experimental systems is important for basic and pre-clinical studies as they allow investigation into the molecular mechanisms of carcinogenesis and the testing of therapies [4]. Most in vitro studies employ the 70 or so well-characterized available breast cancer cell lines including MCF-7, MDA-MB-231, T-47D and ZR-75-30 cells that have been established over the past 30 years. These cell lines were derived from tumor metastases—especially aspirate or pleural effusions and not from primary breast tumors and are from Caucasian or African–American patients [5–7]. Although breast cancer cell lines are easy to handle and replace from frozen stocks, they are prone to genotypic and phenotypic drifts during continuous culture [8, 9]. As such, they may not always appropriate, especially for studies in other ethnic groups. Establishing new breast cancer cell lines representative of the Chinese population can provide a better overall understanding of the etiology and molecular pathogenesis of breast cancer.

To this end, we established three new breast cancer cell lines: ZJU-0725 and ZJU-1127 were derived from invasive ductal carcinoma tissue of one female Chinese patient, whereas ZJU-0327 was derived from another patient. Herein, we describe the characterization of these cell lines in terms of cell morphology, ultrastructure, DNA content, population doubling time (PDT), tumorigenicity, invasive potential, therapeutic sensitivity, protein expression, mycoplasma contamination, TP53 mutation, status karyotype, breast cancer biomarker expression and STR authentication.

## Methods

### Cell lines from cell banks

We used three panels of breast cancer lines as controls—i.e., Normal Phenotype Group (HBL-100 and MCF-10A), Luminal Phenotype Group (MCF-7, T-47D, and Sk-Br_3_), and Basal-Like Group (BT-549 and MDA-MB-231) obtained from the Cell Bank of Type Culture Collection of the Chinese Academy of Sciences (Shanghai, China) in early 2016. The cells were authenticated by DNA-fingerprinting in the Cell Bank at a regular basis. HBL-100 was maintained in Roswell Park Memorial Institute (RPMI) 1640 medium (Invitrogen, Carlsbad CA, USA) with additive [10% fetal bovine serum (FBS) (Gibco, Grand Island, NY, USA), 1% penicillin/streptomycin (Invitrogen)], and 1.5 g/l NaHCO_3_, 2.5 g/l Glucose, and 0.11 g/l sodium pyruvate. MCF-10A was maintained in Mammary Epithelial Cell Growth Medium (MEGM) (Lonza, Walkersville, MD, USA). MCF-7 was maintained in Minimal Essential Medium(Invitrogen) with additive, and 1.5 g/l NaHCO_3_, 0.023 IU/ml bovine insulin, and 0.11 g/l sodium pyruvate. T-47D and Sk-Br_3_ were maintained in Dulbecco’s Modified Eagle’s Medium (Invitrogen), with additive, and 1.5 g/l NaHCO_3_. BT-549 was maintained in RPMI 1640 medium with additive, 1.5 g/l NaHCO_3_, 0.11 g/l sodium pyruvate, and 0.023 IU/ml bovine insulin. MDA-MB-231 was maintained in L-15 medium (Jinuo Biomedical Technology, Hangzhou, China) with additive. Cells were cultured in a humidified 5% CO_2_ incubator at 37 °C. The medium was renewed when a color change was observed.

### Cell isolation and establishment of cell lines

Tissue samples were obtained from patients diagnosed with invasive ductal breast carcinoma, who underwent mammary gland excision at Sir Run Run Hospital of Zhejiang University and were processed for primary culture as previously described [10]. Briefly, after removing normal tissue, necrotic areas, and blood clots, the surgically resected specimens were immediately immersed in cold, triple-antibiotic phosphate-buffered saline (PBS) containing 1% penicillin/streptomycin and amphotericin B (Invitrogen). The sample was then washed three times in antibiotic PBS, minced with scissors into small pieces of 1–3 mm^3^ in a sterile dish containing RPMI 1640, and then transferred to Falcon Cell Strainers (70 μm) (BD Biosciences, Franklin Lakes, NJ, USA) to obtain a single-cell suspension. The cell pellet was resuspended RPMI 1640 medium supplemented with additive, and then seeded in 60-mm Petri dishes in a humidified 5% CO_2_ incubator at 37 °C. After 2 days later, floating cells were washed away when the medium was renewed. Cancer-associated fibroblasts (CAFs) were removed by scraping using a Corning Small Scraper (Corning Inc. Corning, NY, USA). Cancer cells were harvested by treatment with 0.25% trypsin–EDTA (Invitrogen) and subcultured. The cells were sampled at intervals and stored in liquid nitrogen.

### Transmission electron microscopy (TEM) examination

For ultrastructural analysis, cells at passage (P)72 were fixed with 1.2% glutaraldehyde and 2.0% glutaraldehyde for 1 h at 4 °C, post-fixed in 1% osmium tetroxide dehydrated in a graded series of alcohol, and embedded in resin. Ultra-thin sections were cut, stained with uranyl acetate and lead citrate, and visualized under a HT7700 microscope (Hitachi, Tokyo, Japan).

### Evaluation of culture purity and cell cycle status by flow cytometry

CK-pan is a widely used epithelial maker [11]. ZJU-0327, ZJU-0725, and ZJU-1127 cells at P48–55 were collected, washed three times in PBS, fixed for 15 min in 4% formaldehyde, permeabilized in 90% methanol for 30 min at 4 °C, blocked in 10% normal goat serum, and then incubated in PBS containing 0.5% bovine serum albumin (incubation buffer) and anti-cytokeratin (CK) (pan)-fluorescein isothiocyanate antibody (Cell Signaling Technology, Danvers, MA, USA) for 1 h at room temperature. The cells were washed twice with incubation buffer and counted by flow cytometry on a FACSacn instrument (BD Bioscience).

Normal human lymphocytes were used as an internal standard for cell cycle analysis. ZJU-0327, ZJU-0725, ZJU-1127, and MCF-7 in exponential growth phase were processed according to a standard cell cycle staining protocol (Multi Sciences, Hangzhou, China) and then sorted by flow cytometry. The proliferation index (PI) was calculated as (G2/M + S)/(G0/G1 + S + G2/M), and the S-phase fraction (SPF) was calculated as S ÷ (G0/G1 + S + G2/M) to determine the percentages of proliferating and S-phase cells, respectively.

### Cell Counting Kit (CCK)-8 cell growth assay

The growth kinetics of ZJU-0327, ZJU-0725, and ZJU-1127 cells at P40 were examined with the CCK-8 assay (Dojindo Laboratories, Kumamoto, Japan). Cells were seeded in 96-well plates at a density of 5 × 10^3^ and 1 × 10^4^ cells/well as six replicates in 0.2 ml culture medium, with medium containing no cells serving as a control. The growth rate was evaluated at exactly 12 h intervals for 72 h by measuring absorbance at 450 nm. Growth curves were plotted, and PDT (population doubling time) was calculated using the formula PDT = 0.693t/ln (Nt/N0), where t is culture time, N0 is initial absorbance, and Nt is absorbance at time t.

### TP53 mutation screening

To screen for TP53 mutations, total DNA was extracted from three primary breast cancer cell lines using a DNeasy Blood and Tissue kit (Qiagen, Valencia, CA, USA) according to the manufacturer’s instructions. Total DNA was amplified polymerase chain reaction (PCR) using a primer set containing exons 1–11 of TP53 (Table 1) and Taq-polymerase. Gel-purified products were sequenced using the BigDye Terminator v.3.1 Sequencing kit (Applied Biosystems, Foster City, CA, USA).

**Table 1:** Primer sequences for amplification of TP53 exons [13]

Target	Forward primer (5′→3′)	Reverse primer (5′→3′)	Product (bp)	Codon position
Exon 1	GTCGGCGAGAATCCTGACT	CCAACAATGCAACTCCTATGAT	481	0
Exon 2	TCAGACACTGGCATGGTGTT	GGGGACAGCATCAAATCATC	498	1–25
Exon 3	TCAGACACTGGCATGGTGTT	GGGGACAGCATCAAATCATC	498	25–32
Exon 4	GGGACTGACTTTCTGCTCTTGT	GCCAAAGGGTGAAGAGGAATC	543	33–125
Exon 5	GTTTCTTTGCTGCCGTCTTC	TTCCTTCCACTCGGATAAGATG	387	126–187
Exon 6	TACAAGCAGTCACAGCACATGA	GGTCAAATAAGCAGCAGGTTAAGA	370	187–224
Exon 7	GTGAAACCCCGTCTCTACTGAA	GAGGAGAAGCCACAGGTTAAGA	588	225–261
Exon 8	GGAGTAGATGGAGCCTGGTTTT	GTTGGGCAGTGCTAGGAAAG	328	261–307
Exon 9	GGTAAGCAAGCAGGACAAGAAG	TACAACCAGGAGCCATTGTCTT	313	307–331
Exon 10	CATGTTGCTTTTGTACCGTCAT	TGGATACACTGAGGCAAGAATG	408	332–367
Exon 11	AACATATTTGCATGGGGTGTG	CCAGTCTCCAGCCTTTGTTC	1580	367–394

### Cytogenetics analysis

The karyotype of ZJU-0327, ZJU-0725, and ZJU-1127 metaphase cells at P40 was evaluated as previously described [12]. Briefly, cells were exposed to 0.05 mg/ml colcemid for 6 h, incubated in 0.075 M KCl solution at 37 °C for 40 min, and fixed with a mixture of methanol and glacial acetic acid (3:1, v/v). Drops of cell suspension were placed on cold slides and stained with the Remel Gimesa Plus Stain kit (Thermo Fisher Scientific) for 10 min. Samples were analyzed by trypsin Giemsa-banding, and results are expressed according to the recommendations of the International System for Human Cytogenetic Nomenclature (ISCN) (1985).

### Mycoplasma detection by PCR

Culture medium was collected and the absence of mycoplasma was verified with the PCR Mycoplasma Test kit (HuaAn Biotech, Hangzhou, China) according to the manufacturer’s instructions. DNA fragments were visualized under ultraviolet illumination.

### Phenotypic characterization of cell lines by western blotting

Proteins samples of Normal Phenotype Group, Luminal Phenotype Group, Basal-Like Group and Established Group cells (ZJU-0327, ZJU-0725, and ZJU-1127) were subjected to sodium dodecyl sulfate–polyacrylamide gel electrophoresis (Bio-Rad) and transferred to a polyvinylidene difluoride membrane (Millipore, Billerica, MA, USA) that was blocked in 0.1% Tween-20 in Tris-buffered saline (TBS) containing 5% non-fat dry milk(BD Biosciences) for 2 h at room temperature, and probed overnight with primary antibodies against Ki-67, HER2, progesterone receptor (PR) A/B, estrogen receptor (ER)-α, keratin-18, Forkhead box (FOX)A1/hepatocyte nuclear factor (HNF)3α, anterior gradient homolog (AGR)2, epidermal growth factor receptor (EGFR), secreted protein acidic and rich in cysteine (SPARC), vimentin, caveolin 1 and 2, cluster of differentiation (CD) 44, signal transducer and activator of transcription (STAT)3α/β, octamer-binding protein (OCT)4, (sex-determining region Y)-box (SOX)2, Nanog, CD146, vitamin D3 receptor (VDR), and glyceraldehyde 3-phosphate dehydrogenase (GAPDH) (all 1:1000, from Cell Signaling Technology, Danvers, MA, USA). After three 5-min times of washes with 0.1% Tween-20 in TBS, the membrane was incubated with diluted horseradish peroxidase (HRP)-conjugated anti-rabbit secondary antibody (1:2000, Cell Signaling Technology), and imaged with a Tanon 5200 Multi scanner (Tanon, Shanghai, China) after processing with the EZ-ECL kit (Biological Industries, Kibbulz Beit-Haemek, Israel).

### Evaluating sensitivity to chemoradiotherapy and radiation

ZJU-0725 and ZJU-1127 cells at P70 were subjected to chemoradiotherapy [cyclophosphamide, carboplatin, epirubicin HCl, 5-fluorouracil (FU), docetaxel, and paclitaxel (Selleck Chem, Houston, Texas, USA)]; and ZJU-0725 and ZJU-1127 cells at P75 and Sk-Br_3_ cells were exposed to 0, 2, 5, 10, or 15 Gy radiation for 72 h. Cells were seeded at a density of 5 × 10^3^ in 96-well plates, with six replicates per treatment. Cell viability following treatment was evaluated by incubating with CCK-8 reagent for 2.5 h and then measuring the absorbance. Inhibition rate (IR) was calculated with the formula IR = 100 − (mean absorbance of test well/mean absorbance of control well) × 100%.

### Tumorigenicity in nude mice

Female athymic nude mice (BALB/C nu; 4–6 weeks old) (SLAC Laboratory Animals Company, Shanghai, China) were used for heterotransplantation experiments. Animal were maintained in laminar flow cabinets under specific pathogen-free conditions. ZJU-0327, ZJU-0725, and ZJU-1127 cells (1 × 10^7^) at P50 resuspended in 0.1 ml PBS were subcutaneously injected into the right flank of each mouse (n = 4 per group). Tumor diameter was recorded every 5 days. On the last day, tumor tissue was excised, fixed in 10% formalin, embedded in paraffin, and processed for routine histopathologic examination by hematoxylin and eosin (H&E) staining.

### In vitro invasion assay

The invasive potential of ZJU-0327, ZJU-0725, and ZJU-1127 cells at P53 was evaluated using a transwell chamber with an 8.0-μm pore membrane filter (Corning Inc. Corning, NY, USA) coated with 0.3 g/l Matrigel (BD Biosciences). Cells were added to the upper compartment of the chamber, while the lower compartment was filled with 600 μl culture medium. After 48 h, the cells on the top membrane surface were removed, and those that invaded the Matrigel layer on the lower membrane were fixed in methanol for 15 min, stained with 0.1% Crystal Violet for 15 min, and immersed several times in water, and air dried, and sealed with resin. Cells in 10 randomly selected fields were counted under a microscope.

### H&E staining and immunohistochemistry

Immunohistochemical analysis was carried out using paraffin-embedded sections of tumor xenograft samples from mice. The specimen were cut into 4-μm-thick sections, that were mounted on poly-l-lysine-coated slides, dried overnight at 37 °C, deparaffinized in xylene, rehydrated through a graded series of alcohol, and stained with H&E. Immunohistochemical analysis was performed using a standard avidin–biotin technique after a microwave antigen retrieval step in which slides were heated in a pressure cooker containing 10 mM sodium citrate (pH 6.5) for 10 min. Endogenous peroxidase was deactivated by treatment with 3% H_2_O_2_ for 5 min, blocked with 10% normal goat serum for 30 min at room temperature, and probed overnight at 4 °C with primary antibodies against ER-α, PR, HER2, E-cadherin, CK5/6, EGFR, p120, and Ki-67 (Dako, Carpinteria, CA, USA). The following day, slides were incubated with poly-HRP secondary antibodies for 1 h in the dark at room temperature. Immunodetection was performed using 3,3′-diaminobenzidine substrate kit. Sections were counter-stained with hematoxylin to visualize nuclei.

### Verification of cell lines by ATCC

Cells were authenticated according to the protocol of the ATCC (Manassas VA, USA) based on 17 STR loci plus the gender-determining locus, amelogenin. Samples were processed with an ABI Prism 3500 × 1 Genetic Analyzer, and data were analyzed using GeneMapper IDX v.1.2 software (Applied Biosystems). Appropriate positive and negative controls were used, and the results were confirmed against ATCC and Deutsche Sammlung von Mikroorganismen und Zelkulturen (DSMZ) databases for each sample submitted (ATCC Sales Order No. SO0146233).

### Statistical analysis

Data were analyzed using Prism software (GraphPad Inc., LaJolla, CA, USA) and are presented as mean ± standard deviation of at least three independent experiments. Differences with a P value of < 0.05 were considered statistically significant.

## Results

### Primary cell culture and morphological characterization

**Fig. 1:**
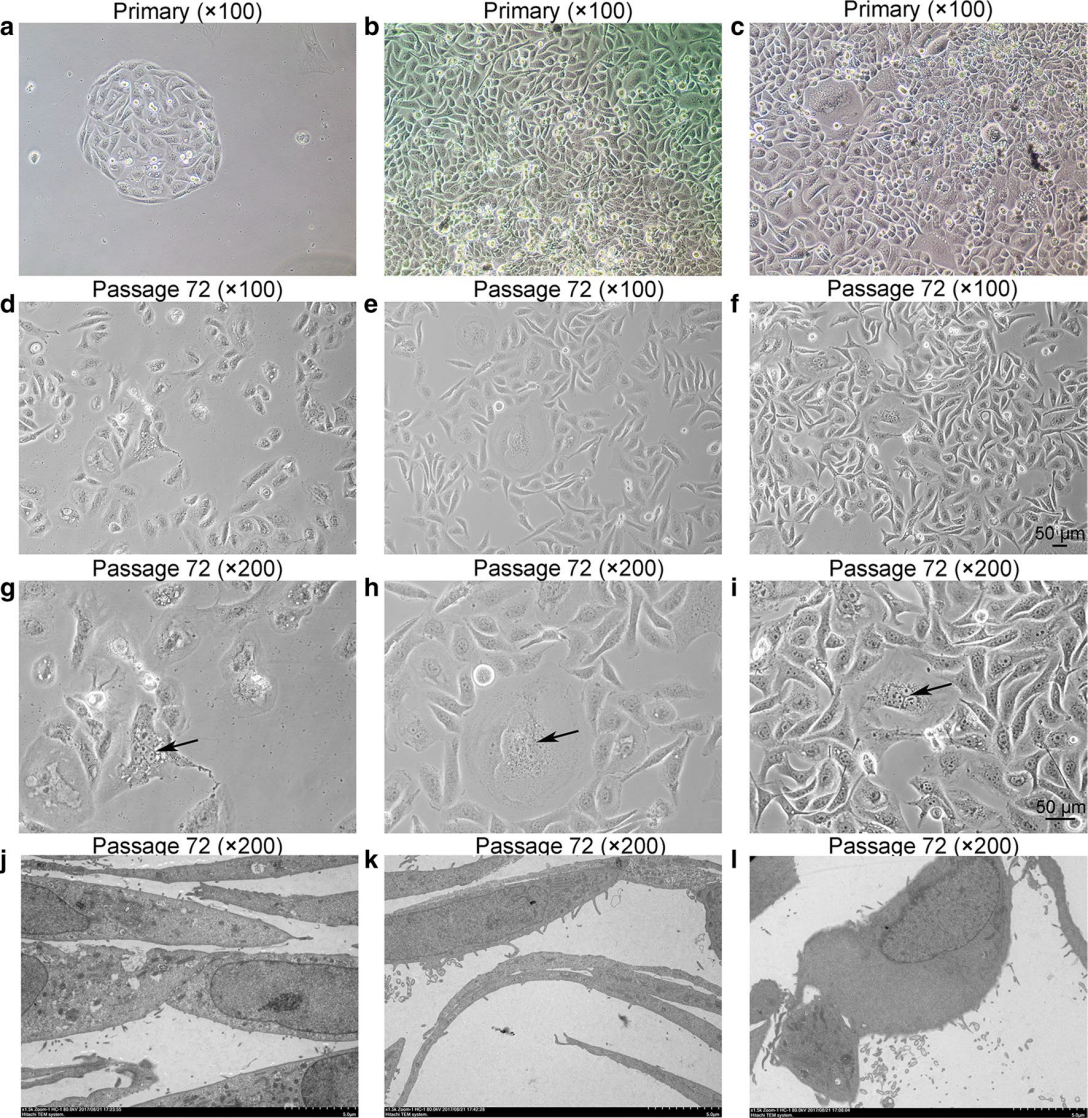
Phase-contrast microscopy and Transmission electron micrograph of cultured ZJU-0327, ZJU-0725 and ZJU-1127 cells. (**a**–**c**, ×100) ZJU-0327, ZJU-0725, and ZJU-1127 cells grew directly from explant 48 h after seeding. (**d**–**f**, ×100; **g**–**i**, ×200) At P72 of ZJU-0327, ZJU-0725, and ZJU-1127cells formed a monolayer and exhibited typical characteristics of malignancy, including numerous mitotic bodies, a high nuclear-to-cytoplasm ratio, prominent nucleoli, occasional multinucleated giant cells (indicated by the arrows), cytoplasmic vacuoles. (**j**–**l**, ×5000) ZJU-0327, ZJU-0725, and ZJU-1127 cells revealed identical ultrastructural features including desmosomes, a sunken nuclear membrane, tonofilaments, cytoplasmic inclusions, and numerous organelles in the cytoplasm

The ZJU-0327, ZJU-0725, and ZJU-1127 cell lines were deposited in the China Center for Type Culture Collection (CCTCC) (Nos. C2017173, C2017171, and C2017172, respectively). ZJU-0327 was derived from one female breast infiltrating ductal carcinoma patient aged 63 years old with axillary lymph node metastases (1 +/15), whereas ZJU-0725 and ZJU-1127 were derived from another patient aged 65 years old with the same diagnosis (1 +/19). To establish primary breast cancer cell lines, original cultured cells were subcloned at a ratio of 1:6, and the epithelial cell cluster was marked. Fibroblasts were removed by scraping method. Established cells grew as an adherent monolayer and were spindle shaped with epithelial and malignant characteristics, although ZJU-0725 cells were more narrow than cells in the other two lines (Fig. 1a–i). All cells were obtained from primary tissue and maintained in RPMI 1640 medium with additive, and were propagated for more than 100 passages, during which there were no changes in cell shape or growth pattern.

### Ultrastructural analysis

Cell ultrastructure was examined by TEM. The cells showed typical epithelial characteristics including an abundance of rough and smooth endoplasmic reticula, thin and elongated mitochondria, polyribosomes, large and irregular nuclei, and a sunken nuclear membrane. There were many microvillus-like protrusions on the cell surface, and desmosomes at intercellular junctions as well as microfilament bundles in the cytoplasm. On the other hand, no secretory bodies or well-developed Golgi apparatus were observed (Fig. 1j–l).

### Purity, content DNA and and PDT

**Fig. 2:**
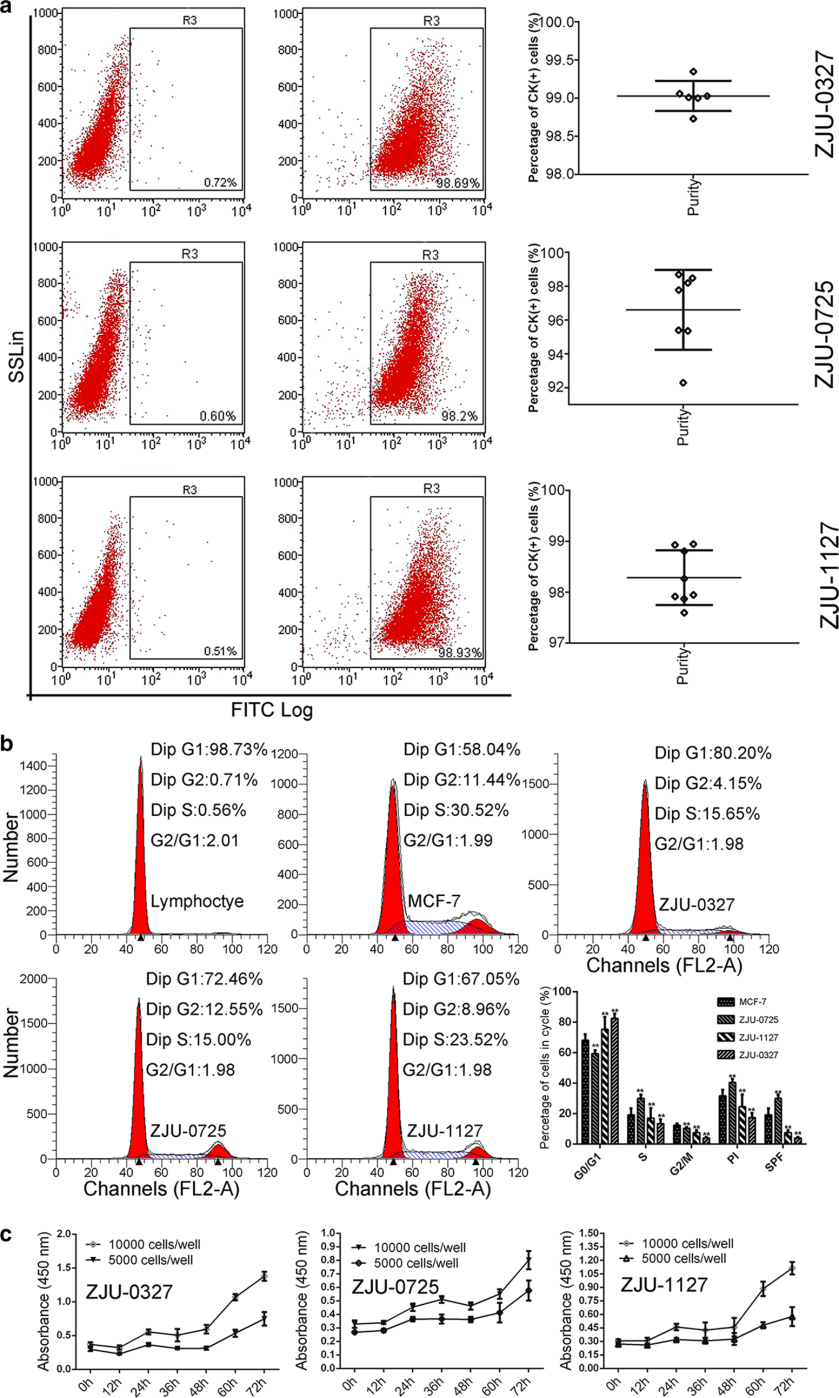
Purity, DNA content, and doubling time of ZJU-0327, ZJU-0725, and ZJU-1127 cell lines. **a** The purity was evaluated by flow cytometry and statistical analysis. **b** DNA content and cell cycle distribution of normal lymphocytes and MCF-7, ZJU-0327, ZJU-0725, and ZJU-1127 cells were evaluated by flow cytometry and statistical analysis. **c** Growth rate of ZJU-0327, ZJU-0725, and ZJU-1127 cells was determined with the CCK-8 assay

The purity of the ZJU-0327, ZJU-0725, and ZJU-1127 cell lines was 97.32% ± 1.53%, 98.46% ± 0.50% and 99.03% ± 0.120%, respectively (Fig. 2a). The DNA content was determined by flow cytometry. The G0/G1 phase was diploid, the S phase was polyploid between diploid and tetraploid, and the G2/M phase was polyploid, indicating that all three cell lines along with MCF-7 cells were aneuploid, in contrast to diploid lymphocytes. The PI and SPF of ZJU-0725 cells were higher whereas these values for ZJU-0327 and ZJU-1127 cells were lower than for MCF-7 cells (P < 0.05; Fig. 2b). PDT was determined with CCK-8 assay. The PDT of ZJU-0327 and ZJU-1127 cells was about 18 h; however, ZJU-0725 cells grew more slowly, with a PDT of 57.5 h that was unrelated to initial seeding density (Fig. 2c).

### TP53 gene mutations and mycoplasma contamination

**Fig. 3:**
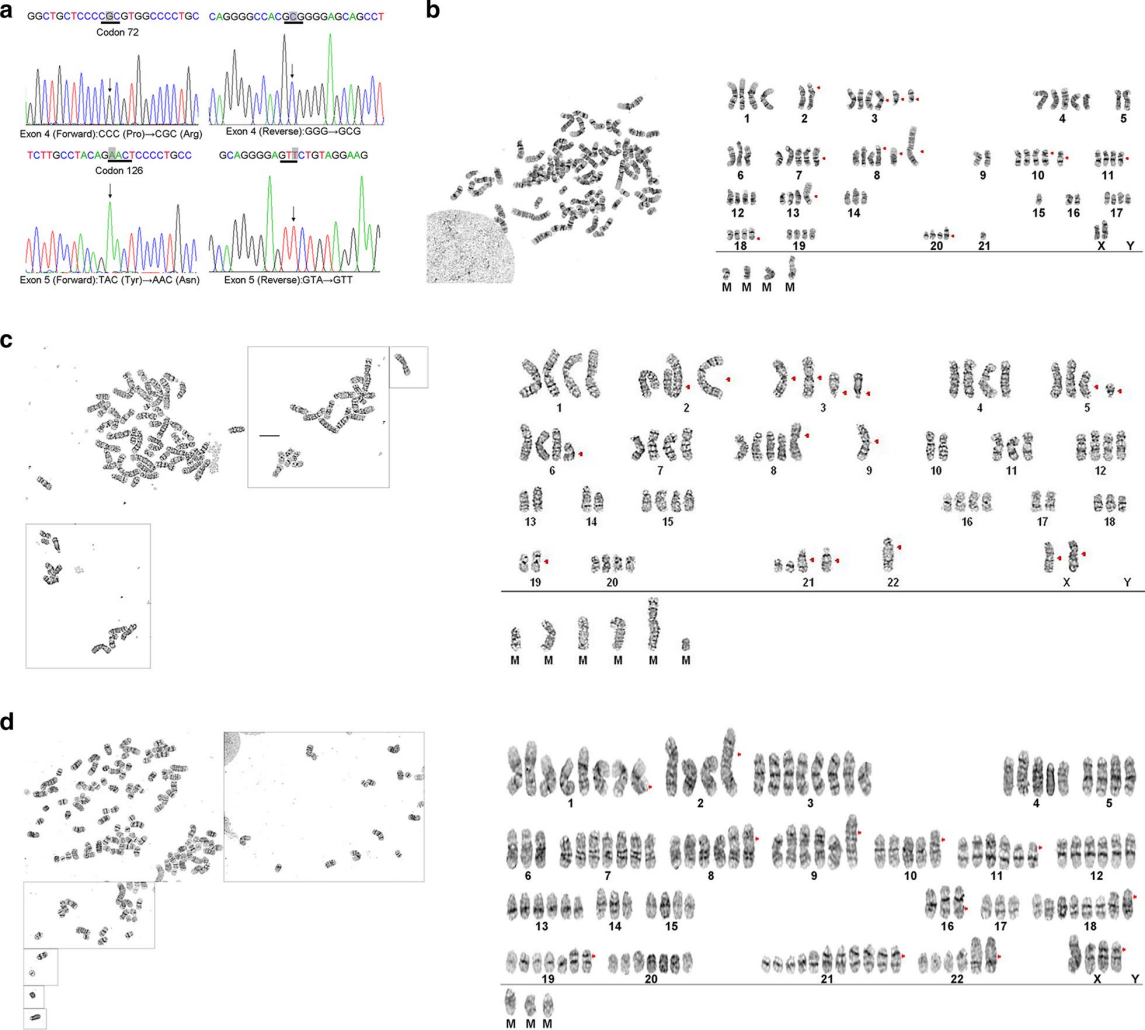
TP53 mutation screening and conventional cytogenetics. **a** SNP in exon 4 (forward and reverse) and missense mutations in exon 5 (forward and reverse). **b** Representative karyotype of one ZJU-0327 cells at the P35: 81. Red arrows point to der (2) t (2;9) (p23;q21), +i(3) (q10), +del (3) (q13.1)×2, +4, +5, +7, +8, +add (8) (p23), +der (8) t (8;?) (p12;?), −9×3, −10, +11×2, +12, +add (14) (p13), +15, −16, −17, +der(19) t (19;?) (q13.3;?), +der(21) t (21;?) (q22;?), −22×2 [CP3]. **c** Representative karyotype of ZJU-0725 cells at the P25: 78. Red arrows point to der(X)t(X;?)(q21;?),i(X)(q10),−2,add(2)(p25),der(2)t(2;?)(p11.2;?),del(3)(q12)×2,+i(3)(q10)×2,del(5)(q11.2),−6,i(6)(p10),+der(8)t(8;?)(p12;?),−9×3,der(9)t(9;?)(p13;?),−10×2,−11,−12,−13×2,−14×2,−17×2,−18,−19×2,add(19)(p13.3),add(21)(q22)×2,−22×3,add(22)(q13),+mar1,+mar2,+mar3,+mar4,+mar5[cp10]. **d** Representative karyotype of ZJU-1127 cells at the P23: 133 der(X)t(X;?)(p11.2;?)×3,i(X)(q10),+der(1)t(1;9)(p10;q10)×4,−2,add(2)(p25),+i(3)(q10)×8,−5,−6×2,+7×2,+der(8)t(8;?)(p12;?),+der(9)t(9;?)(p13;?)×5,der(10)t(10;?)(q22;?),+del(11)(p11.2)×2,+12,+13,−14×2,−15,−16×2,der(16)t(16;?)(q12;?)×2,−17×2,+der(18)t(18;?)(q11.2;?)×2,+add(19)(p13.3)×2,add(21)(p13),+add(21)(q22)×6,+add(22)(q13)×2,+mar1,+mar2,+mar3[1]

**Fig. 4:**
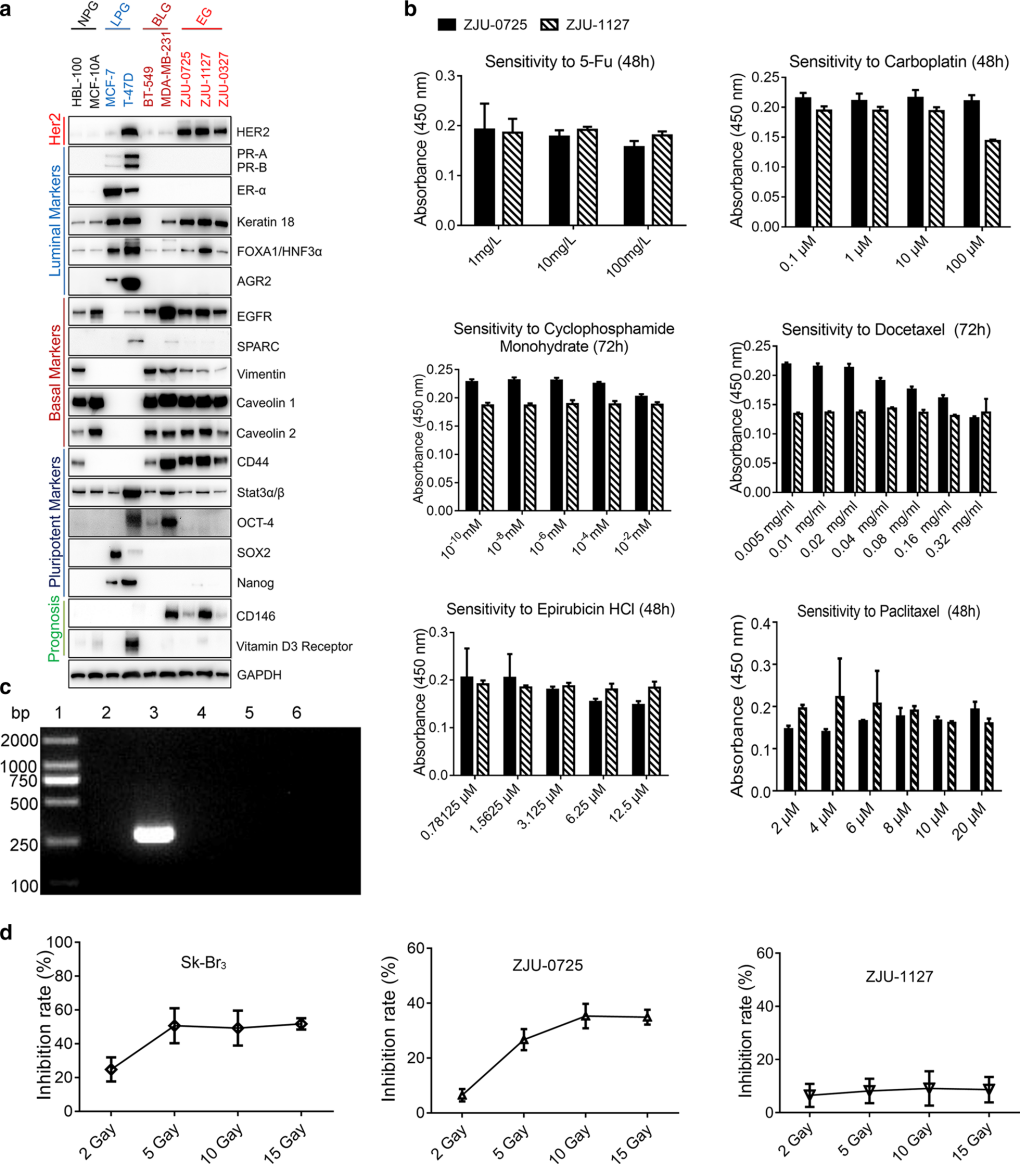
Analysis of phenotype, drug sensitivity, mycoplasma contamination, and radiation sensitivity. **a** Protein expressing patterns of ZJU-0327, ZJU-0725, and ZJU-1127 cells were more similar to those of the basal-like phenotype group than the normal and luminal phenotype groups and HER2 over expression, as determined by western blotting. **b** Absence of mycoplasma contamination in the negative control sample and ZJU-0327, ZJU-0725, and ZJU-1127 cell lines, as determined by PCR. **c** ZJU-0725 and Sk-Br_3_ but no but ZJU-1127 were sensitive to radiation (different X-ray doses for 72 h), **d** cyclophosphamide, carboplatin, 5-FU, and paclitaxel showed slight inhibition of ZJU-0725 growth but had no effect on ZJU-1127

Mutations in the coding region of the TP53 gene were detected by sequencing. All three cell lines harbored mutations in the same location namely, a single nucleotide polymorphism (SNP) from proline (CCC) to arginine (CGC) at codon 72 of exon 4 and a missense mutation from tyrosine (TAC) to asparagine (AAC) at codon 126 of exon 5 (Fig. 3a). The three lines were also tested for the presence of mycoplasma at regular intervals and were found to be free of mycoplasma contamination (Fig. 4c).

### Chromosome analysis

A karyotype analysis revealed that, the three cell lines had abnormalities in chromosome number and structure. ZJU-0327 was near tetraploid, with a modal number of chromosomes of 80–82. Trypsin-Giemsa banding revealed a deletion in chromosome 2 and additional chromosomes 8 and 14, while many derivative chromosomes (2, 8, 19, and 21) resulting from unbalanced translocation with materials of known or unknown origin were observed. There were also additions in chromosomes 1, 3–8, 11–15, and 18–21, and loss of chromosomes 9, 16, and 22. ZJU-0725 was also near tetraploid, with a modal number of chromosomes of 78–85. Typsin-Giemsa banding revealed a double-deletion of chromosomes 3 and 5; additional chromosomes 2, 9, 21, and 22; derivative chromosomes of X, 2, 8, and 9; additions in chromosomes 1–8, 11, 12, 15, 16, 18, 20, and 21; and loss of chromosomes 9 and 22. ZJU-1127 was aneuploid with near pentaploid to hexaploid complement, with chromosome number ranging from 128 to 133. Trypsin-Giemsa banding revealed a deletion chromosomes 11; additional chromosomes 2, 19, 21, and 22; derivatives of all chromosomes including X; and no chromosome loss. Unknown derivative marker chromosomes were also observed in the three cell lines (Fig. 3b–d).

### Phenotypic analysis by western blotting

To characterize the phenotype of the three breast cancer cell lines by western blotting, cells were divided into Normal Phenotype Group, Luminal Phenotype Group, Basal-Like Group, and Established Group cells. HER2 was expressed in Luminal Phenotype Group and Established Group. PR, ER-α, AGR2, SPARC, SOX2, Nanog and VDR were expressed only in Luminal Phenotype Group. Keratin-18, FOXA1/HNF3α, and STAT3α/β were expressed at similarly high levels in Luminal Phenotype Group and Established Group, but were weakly expressed in Normal Phenotype Group and Basal-Like Group. EGFR, vimentin, caveolin1 and 2, and CD44 were expressed in Normal Phenotype Group, Basal-Like Group, and Established Group, but not in Luminal Phenotype Group. OCT4 was expressed in Luminal Phenotype Group and Basal-Like Group, but not in Normal Phenotype Group and Established Group. CD146 was expressed in Basal-Like Group and Established Group. Thus, Established Group cells exhibited the Basal/HER2 phenotype, which is associated with poor prognosis (Fig. 4a).

### Sensitivity to chemoradiotherapy and radiation treatment

Docetaxel and epirubicin HCl showed weaker inhibitory effects against ZJU-0725 as compared to ZJU-1127. On the other hand, 5-FU, paclitaxel, cyclophosphamide monohydrate and carboplatin had no effect on ZJU-0725 and ZJU-1127 cell proliferation (Fig. 4b). When cells were exposed to different X-ray doses for 72 h, the viability of ZJU-025 and Sk-Br_3_ cells was reduced at 5 Gy, whereas, ZJU-1127 cells showed no change in viability at any dose (Fig. 4d).

### Invasivenss and tumorigenicity

**Fig. 5:**
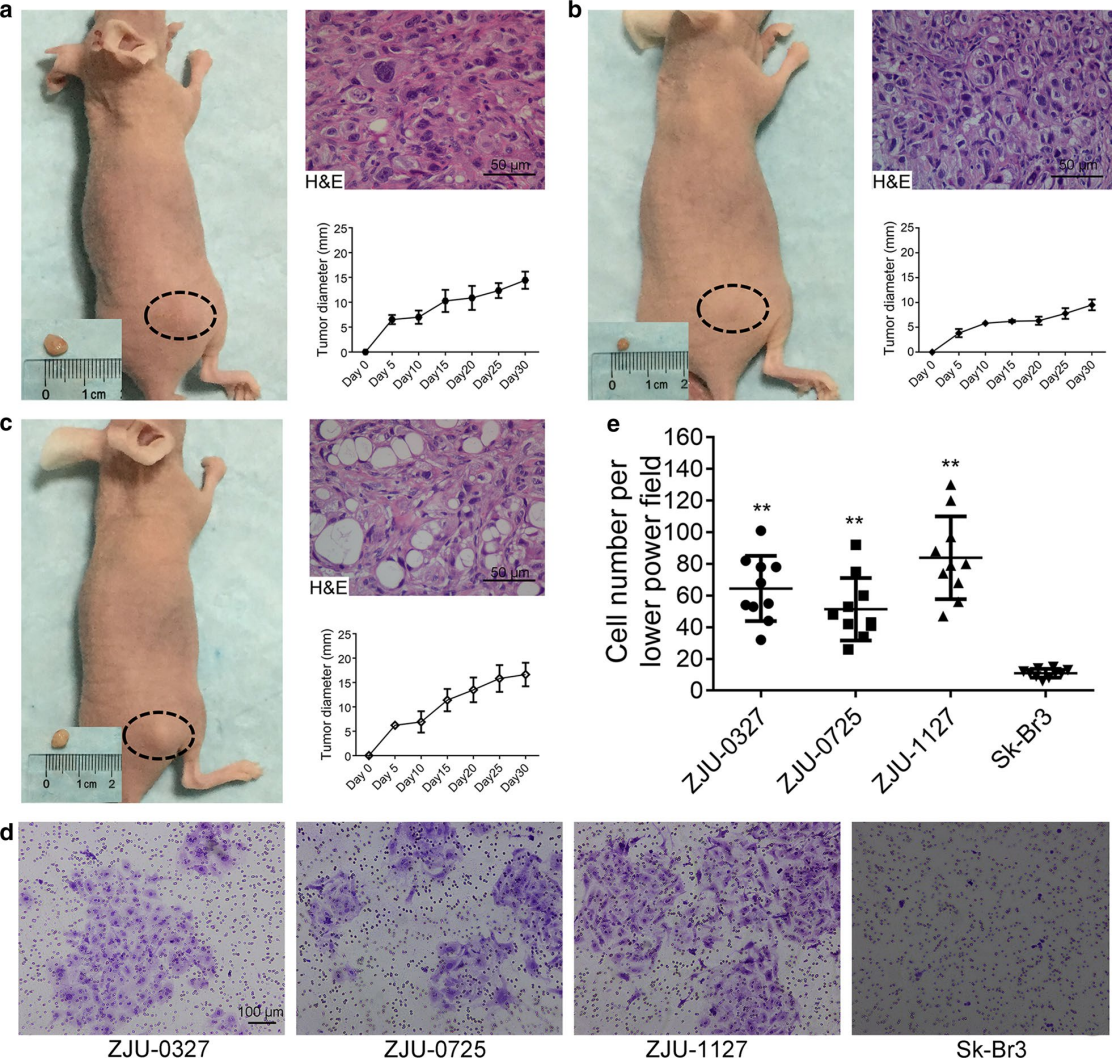
Tumorigenicity in immunodeficient mice and migratory and invasive potential, as determined with the transwell assay. Tumor formation in immunodeficient mice (tumor size indicated by the ruler, and corresponding H&E staining (×200) and tumor growth curves of ZJU-0327 (**a**), ZJU-0725 (**b**) and ZJU-1127 (**c**) cells. Statistical comparison of invasive cells (**d**) and representative micrographs of ZJU-0327, ZJU-0725, ZJU-1127, and Sk-Br_3_ cells (×100) (**e**)

To assess in vivo tumorigenicity, we carried out xenograft transplantation of the three cell lines in BALB/c nu mice. All mice developed tumor after subcutaneous implantation of the cells, with ZJU-0327 and ZJU-1127 cells exhibiting more rapid growth than ZJU-0725 cells. H&E staining revealed that tumors xenografts had the histological characteristics of poorly differentiated adenocarcinoma (Fig. 5a–c). Lymph node metastasis was not observed. We used the transwell assay to further investigate the invasive potential of the three breast cancer cell lines, and found that ZJU-0327, ZJU-0725, and especially ZJU-1127 cells exhibited greater invasiveness than Sk-Br_3_ cells (Fig. 5d, e).

### Immunohistochemical analysis of original breast tumor and xenograft

**Fig. 6:**
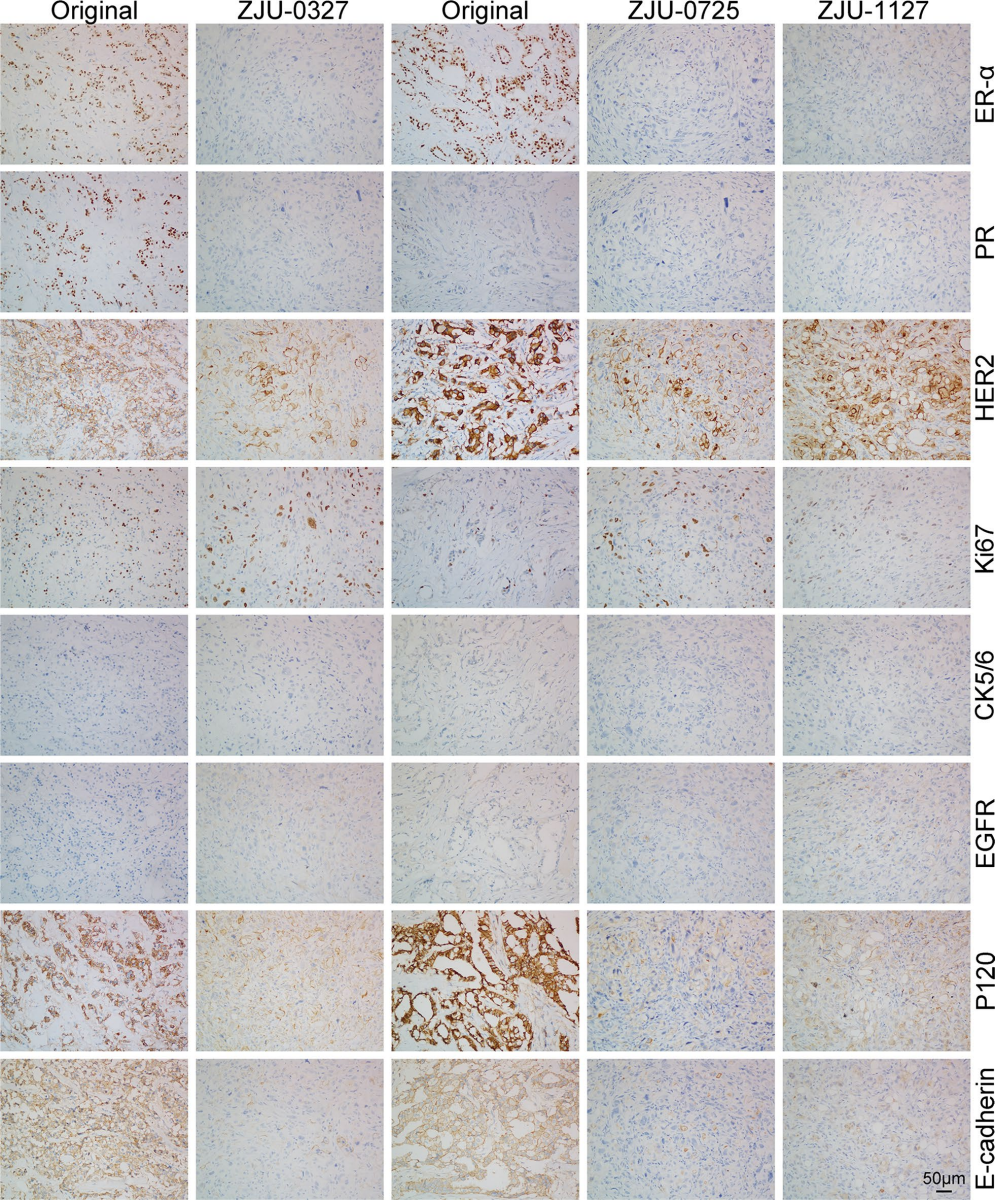
Immunophenotype of ZJU-0327, ZJU-0725 and ZJU-1127 cells in sections from original and xenografted tumors. On the sections of ZJU-0725 and ZJU-1127, strongly positive for ERα, Her2, EGFR, P120 and E-cadherin in original tumors and Her2, EGFR, P120 and E-cadherin in the ZJU-0725 xenografted tumors and Her2, P120 and E-cadherin in the ZJU-0725 xenografted tumors; positive for ki67 in original tumors and ERα and Ki67 in the ZJU-0725 xenografted tumors and ERα, Ki67 and EGFR in the ZJU-1127 xenografted tumors. But negative for PR and ck5/6 in both original and xenografted tumors. On the sections of ZJU-0327, strongly staining for ERα, PR, Her2, P120 and E-cadherin in the original tumors and Her2, P120 and E-cadherin; positive for Ki67 in the original tumors and ERα, ki67 and EGFR. But negative for ck5/6 and EGFR in the original tumors and PR and ck5/6 in the xenografted

Clinical breast tumor specimens and tumor xenografts were immunolabeled for ERα, PR, HER2, Ki67, CK5/6, EGFR, P120, and E-cadherin. ZJU-0725 and ZJU-1127 tumor xenografts showed PR, HER2, Ck5/6, P120, and E-cadherin expression patterns that were identical to those of clinical specimens. EGFR expression was downregulated in ZJU-1127 compared to ZJU-0725 and clinical samples. ZJU-0725 and ZJU-1127 xenografted showed increased Ki67 but decreased ERα levels as compared to patient tumors. HER2 and EGFR were upregulated whereas ERα and PR downregulated in ZJU-0327 cell-derived tumors as compared to clinical samples, whereas P120, CK5/6, Ki67, and E-cadherin were similarly expressed in both sets of tissue (Fig. 6).

### DNA fingerprinting/STR analysis

STR analysis of ZJU-0327, ZJU-0725, and ZJU-1127 cell lines confirmed that they were of human origin and were distinct from other cell lines in ATCC and DSMZ database (ATCC ID Nos. SATR7097, SATR6985, and SATR 6987 respectively).

## Discussion

In this study we successfully established three primary Basal/HER2+ breast cancer cell lines (ZJU-0327, ZJU-0725, and ZJU-1127), two of which originated from a single patient. The cell lines were derived from poorly differentiated in situ breast invasive ductal carcinoma of untreated female patients from China. In both cases, there was no family history of breast carcinoma, and no other factors that are known to have contributed to the disease.

Many studies have described methods for establishing breast cancer cell lines, including differential trypsinization [12–16], adherence [17], tissue inoculation [12, 18–20], geneticin treatment [21, 22], and differential centrifugation [23], among others. We used the cell scraping method. The three cell lines were established approximately 7 weeks after initial seeding, and were sensitive to 0.25% trypsin–EDTA like other established breast cancer cell lines. We therefore deemed differential trypsinization unsuitable for to eliminating CAFs. The cell lines were passaged for 10 months and subcultured for more than 110 passages without obvious changes in morphology or proliferative potential after cryopreservation and resuscitation (Fig. 1a–i). The cells also retained ultra-structural features consistent with their malignant epithelial origin (Fig. 1j–l), including numerous cytoplasmic organoids, desmosomes, and tonofilaments [19, 21].

The purity of ZJU-0327, ZJU-0725, and ZJU-1127 cells ranged from about 97% (Fig. 2a), possibly reflecting epithelial-to-mesenchymal transition [24, 25]. Similar to MCF-7 cells, ZJU-0327, ZJU-0725, and ZJU-1127 were polyploid. ZJU-0725 had the highest PI and SPF values indicating the highest proliferative potential, followed by MCF-7, ZJU-1127, and ZJU-0327 (Fig. 2b). PDT differs among breast cancer cell lines, indeed, in our cells the doubling times ranged from 48 to 60 h irrespective of initial seeding density (Fig. 2c). One reason for the variability is differences in proliferation rate [26].

Mutations in tumor suppressor genes including TP53 are often detected in several cancers including breast cancer [27, 28]. We screened all coding regions (11 exons) of the TP53 gene in the three cell lines and found identical mutations. Interestingly, the CCC/CGC (Pro/Arg) SNP (rs1042522, http://www.ncbi.nlm.nih.gov/snp/?term=rs104522) was present in codon 72 of exon 4, this is common in human cancers and is considered non-pathogenic. Codon 126 of exon 5, which is located in the sequence-specific DNA-banding region, harbored a TAC (Try) to AAC (Asn) missense mutation (MUT_ID:1320) (http://www.p53.iarc.fr) (Fig. 3a), which could result in loss of the DNA binding function and pathogenesis.

Chromosomal abnormalities can lead to the development of diseases including cancer. Our three breast cancer cell lines showed abnormal karyotypes, including increases in chromosome number as well as chromosomal breaks and translocations (Fig. 3b–d). Although ZJU-0725 and ZJU-1127 were from the same patient, they exhibited different karyotypes. Fractured chromosomes were also observed in ZJU-0327, ZJU-0725, and ZJU-1127, which can explain the observed hyperdiploidy.

Basal/HER2+ breast cancer shows poorer survival than other phenotypes [29, 30]. We examined the expression of HER2, along with luminal (PR, ER-α, keratin 18, FOXA1/HNF3α, and AGR2) [31], basal (EGFR, SPARC, Vimentin, and caveolin1–2), pluripotent (CD44 [32], and Stat3α/β, OCT-4, SOX2, and Nanog [33, 34]) and prognosis (CD146 and VDR) markers by western blotting. CD146 [35], and VDR [36, 37] are associated with poor and excellent prognosis, respectively. We found that ZJU-0327, ZJU-0725, and ZJU-1127 cells exhibited the basal/HER2+ phenotype, including stem-cell-like properties, and the owner of these cell lines has low survival (Fig. 4a). Given the poor prognosis of basal/HER2+ breast cancer, we investigated sensitivity of the cell lines to various therapies ZJU-0725 and ZJU-1127 were resistant to the tested drugs, and while the former cells were sensitive to radiation, the latter were not (Fig. 4b, d). Notably, trastuzumab was in-effective against our cell lines (data not shown).

We investigated the malignant potential of our cell lines in a xenograft model and found that all three cell lines formed tumors in nude mice, with the largest tumors formed by ZJU-0327 and ZJU-1127 cells in 30 days. The different growth patterns observed in vitro and in vivo were likely due to environmental variations. Our histological analysis confirmed that the tumor xenografts were poorly differentiated (Fig. 5a–c). Although the donors had bilateral lymph node metastasis, this was not observed in our model, possibly because the observational period of 30 days was insufficient or because the cells were not implanted into (breast) tissue but were instead injected into the subcutaneous space. Nonetheless, our experiments showed that ZJU-0327, ZJU-0725, and especially ZJU-1127 had higher migratory and invasive potential than Sk-Br_3_ cells (Fig. 5d, e).

We observed differences in protein expression between tumor xenografts and clinical specimens. The expression patterns of CK5/6, P120, and E-cadherin were similar in tissue sections from xenograft and corresponding original tumors. PR and HER2 expression was comparable between ZJU-0725 and ZJU-1127 tumor xenografts and original tumors, whereas Ki67 was similarly expressed in ZJU-0327 cell-derived tumors and clinical samples. We also observed that compared to the original tumors, Ki67 and ER-α immunoreactivity was increased and decreased, respectively, in ZJU-0725 and ZJU-1127, while HER2 and EGFR immunoreactivity was upregulated and ERα and PR were downregulated in ZJU-0327. The difference may be due to a well-differentiated cell subpopulation and/or the presence of factors that increased the differentiation of ZJU-0327, ZJU-0725, and ZJU-1127 cells during the establishment of these cell lines. Expression of the proto-oncogene and receptor tyrosine kinase EGFR was correlated with cell proliferation, survival, migration, and differentiation, and its over-expression can lead to the development and recurrence of breast cancer [38]. We found that EGFR expression was reduced in ZJU-1127 tumor xenografts relative to ZJU-0725 and the original tumors (Fig. 6). The differences in immunoreactivity between ZJU-0725 and ZJU-1127 xenografts may be explained by tumor heterogeneity.

## Conclusions

We established three novel basal/HER2+ breast cancer cell lines from female Chinese patients who were diagnosed with invasive breast cancer. The cell lines recapitulate the malignant characteristics of the primary tumor. These three cell lines can serve as new models for investigating the etiology and molecular pathogenesis for basal/HER2+ breast cancer and for testing novel therapeutics for its treatment.

## Abbreviations

HER2+: human epidermal growth factor receptor (HER)2-positive
STR: short tandem repeat
TP: tumor protein
ATCC: the American Type Culture Collection
PDT: population doubling time
FBS: fetal bovine serum
MEGM: Mammary Epithelial Cell Growth Medium
PBS: phosphate-buffered saline
CAFs: cancer-associated fibroblasts
TEM: transmission electron microscopy
P: passage
CK: cytokeratin
CCK: Cell Counting Kit
PCR: polymerase chain reaction
ISCN: the International System for Human Cytogenetic Nomenclature
TBS: tris-buffered saline
PR: progesterone receptor
ER: estrogen receptor
FOX: Forkhead box
HNF: hepatocyte nuclear factor
AGR: anterior gradient homolog
EGFR: epidermal growth factor receptor
SPARC: secreted protein acidic and rich in cysteine
CD: cluster of differentiation
STAT: signal transducer and activator of transcription
OCT: octamer-binding protein
SOX: (sex-determining region Y)-box
VDR: vitamin D3 receptor
GAPDH: glyceraldehyde 3-phosphate dehydrogenase
HRP: horseradish peroxidase
FU: fluorouracil
IR: inhibition rate
H&E: hematoxylin and eosin
DSMZ: Deutsche Sammlung von Mikroorganismen und Zelkulturen
CCTCC: the China Center for Type Culture Collection
SNP: single nucleotide polymorphism
